# Predictors of acute kidney injury under continuous infusion of vancomycin in critically ill surgical patients

**DOI:** 10.1186/2197-425X-3-S1-A637

**Published:** 2015-10-01

**Authors:** R Chackupurakal, J Reessing, F Wappler, SG Sakka

**Affiliations:** Anästhesie und Operative Intensivmedizin, Kliniken der Stadt Köln gGmbH, Krankenhaus Merheim, Köln, Germany; Anästhesie und Intensivmedizin, Klinikum Oberberg, Kreiskrankenhaus Gummersbach, Gummersbach, Germany

## Introduction

Vancomycin is one of the first antibiotic choices for the treatment of severe gram-positive bacterial infections, especially if due to methicillin-resistant Staphylococcus aureus. Vancomycin is suspected to cause nephrotoxicity, hence continuous vancomycin therapy as an alternative to intermittent administration has been proposed to reduce the risk of developing acute kidney injury (AKI). So far, vancomycin concentration and duration of therapy were identified as the strongest variables associated with the development of AKI during continuous vancomycin therapy [1].

## Objectives

To examine the impact of continuous infusion of vancomycin on renal function in critically ill surgical patients.

## Methods

With approval by our institutional review board, we retrospectively reviewed the data of 139 adult patients (78 men, 61 women; mean age 58 ± 19 years) admitted to the operative intensive care unit between June 2011 and July 2013 who received continuous infusion of vancomycin for more than 48 hours. Renal function was assessed by analyzing serum creatinine levels and urine output from the first day of vancomycin therapy until 72 hours after its discontinuation. Vancomycin concentrations were measured daily in 78 patients and intermittently in 61 patients; the mean vancomycin concentration (C_mean_) and the maximal drug concentration (C_max_) were specifically analyzed. Creatinine clearance was calculated by the Cockcroft-Gault formula. According to the Acute Kidney Injury Network, AKI is defined as an increase in the serum creatinine level of ≥ 0.3 mg/dl or ≥ 50 % from baseline or a daily urine output ≤ 0.5 ml/kg/h. Differences between patients with and without AKI were assessed using a Student's test for independent variables. In the population of patients with AKI, a multivariate analysis was performed. A p-value < 0.05 was considered as statistically significant.

## Results

Nineteen patients (14%) developed AKI during the study period. These patients were significantly older (mean age 67 vs. 57 years), had a higher morbidity and lower creatinine clearance on the first day of vancomycin therapy than those who did not develop AKI. C_max_ was independently associated with the development of AKI.

## Conclusion

In our study, the rate of AKI during continuous vancomycin therapy was lower than in a previous study in critically ill patients treated in a medical-surgical intensive care unit. Our findings should be verified with eligible prospective studies.
